# Assessment of published models and prognostic variables in epithelial ovarian cancer at Mayo Clinic

**DOI:** 10.1016/j.ygyno.2015.01.539

**Published:** 2015-01-22

**Authors:** Andrea Wahner Hendrickson, Kieran M. Hawthorne, Ellen L. Goode, Kimberly R. Kalli, Krista M. Goergen, Jamie N. Bakkum-Gamez, William A. Cliby, Gary L. Keeney, Dan W. Visscher, Yaman Tarabishy, Ann L. Oberg, Lynn C. Hartmann, Matthew J. Maurer

**Affiliations:** 1 Department of Medical Oncology, Mayo Clinic, Rochester MN; 2 Department of Health Sciences Research, Mayo Clinic, Rochester MN; 3 Department of Obstetrics and of Gynecology, Mayo Clinic, Rochester MN; 4 Department of Anatomic Pathology, Mayo Clinic, Rochester MN; 5 Washington University, St. Louis, MO

## Abstract

**Objectives:**

Epithelial ovarian cancer (EOC) is an aggressive disease in which first line therapy consists of a surgical staging/debulking procedure and platinum based chemotherapy. There is significant interest in clinically applicable, easy to use prognostic tools to estimate risk of recurrence and overall survival. In this study we used a large prospectively collected cohort of women with EOC to validate currently published models and assess prognostic variables.

**Methods:**

Women with invasive ovarian, peritoneal, or fallopian tube cancer diagnosed between 2000-2011 and prospectively enrolled into the Mayo Clinic Ovarian Cancer registry were identified. Demographics and known prognostic markers as well as epidemiologic exposure variables were abstracted from the medical record and collected via questionnaire. Six previously published models of overall and recurrence-free survival were assessed for external validity. In addition, predictors of outcome were assessed in our dataset.

**Results:**

Previously published models validated with a range of c-statistics (0.587-0.827), though application of models containing variables not part of routine practice were somewhat limited by missing data; utilization of all applicable models and comparison of results is suggested. Examination of prognostic variables identified only the presence of ascites and ASA score to be independent predictors of prognosis in our dataset, albeit with marginal gain in prognostic information, after accounting for stage and debulking.

**Conclusions:**

Existing prognostic models for newly diagnosed EOC showed acceptable calibration in our cohort for clinical application. However, modeling of prospective variables in our dataset reiterates that stage and debulking remain the most important predictors of prognosis in this setting.

## Introduction

In 2013, ovarian cancer accounted for approximately 22,240 (3%) new cancer cases and 14,030 (5%) cancer deaths in US women [1]. Upon diagnosis, these patients are initially treated with a combination of surgical resection of all visible intra-peritoneal disease (if feasible) and platinum based chemotherapy, administered intravenously or intraperitoneally [2, 3]. Despite initial aggressive treatment, more than 75% of patients experience a recurrence within the first 20 months of therapy completion [3]. Currently, there are no validated biomarkers to predict recurrence.

There have been several attempts to design clinically feasible prognostic models in ovarian cancer [4-9]. In 2001, Clark et al. utilized data from the Ovarian Cancer database of the Imperial Cancer Research Fund (ICRF) in Edinburgh to develop a prognostic model for overall survival (OS) in a cohort of 1189 women with primary epithelial ovarian cancer (EOC) diagnosed between 1984 and 1999. Multivariate analysis found that older age, higher FIGO stage, worse performance status, lower albumin, high grade, greater residual disease and greater (log) alkaline phosphatase were associated with a significantly greater risk of mortality [4]. In 2012, Barlin et al. developed a nomogram to predict the 5-year disease- specific mortality after primary surgery in all stages of primary EOC from a cohort of 478 patients from Memorial-Sloan Kettering. The model was based on seven predictor variables: residual disease after primary surgery, International Federation of Gynecology and Oncology (FIGO) stage, tumor histology, age, albumin, family history suggestive of hereditary breast and ovarian cancer (HBOC) syndrome, and American Society of Anesthesiologists (ASA) physical status [5]. Most recently, Rutten et al. developed a prognostic model for 5-year OS derived from a clinical cancer registry of three centers in the Netherlands [10]. Unlike the two prior models, this model includes women who underwent interval debulking as well. In this cohort, predictors included in the nomogram were age, type of surgery (primary or interval cytoreduction), residual disease, histology, stage, performance status, ascites, and BRCA status with endpoints of disease-specific survival at 1, 3, and 5 years.

Because the majority of ovarian cancers are detected at advanced stage [11], there has also been interest and effort in developing prognostic models solely for advanced EOC (stage III/IV). In 2007, Chi et al. developed a nomogram to predict 5-year disease-specific survival for bulky stage IIIC EOC using a total of 424 patients who underwent a debulking surgery and subsequently received platinum based therapy, using six variables [7]. Shortly thereafter, in one of the largest datasets to date, Teramukai et al. published the PIEPOC (Prognostic Indicator for advanced Epithelial Ovarian Cancer) model for 5- year OS, which was derived from data from 768 women with stage III/IV EOC from 24 institutions in Japan [6]. Of the six factors initially evaluated, four prognostic factors, namely residual tumor size, histologic cell type, performance status and age, remained independently significant. From this, they were able to identify three recurrence risk groups (low, intermediate and high). This study was also validated in a Western European population [12]. A smaller study by Gerestein and colleagues, which included 118 patients with stage IIB-IV EOC from the Ovarian Cancer Database in the Netherlands, developed a nomogram for progression-free survival (PFS) and OS for women who had undergone an initial debulking surgery followed by 6-9 cycles of adjuvant platinum based chemotherapy [8]. In this study, residual disease and preoperative platelet count were predictive of PFS while residual disease, preoperative platelet count and preoperative hemoglobin were predictive of OS.

In this study we validated these previously published models in an external cohort of 701 women with invasive ovarian, peritoneal, or fallopian tube cancer enrolled in a longstanding registry at the Mayo Clinic. We also performed an assessment of predictors of progression-free and overall survival in our cohort to identify the utility of prognostic factors beyond stage and debulking.

## Materials and Methods

### Study population

Patients with invasive ovarian, peritoneal, or fallopian tube cancer diagnosed between January 1, 2000 and December 31, 2011 and prospectively enrolled into the Mayo Clinic Ovarian Cancer registry were identified. The study was approved by the Mayo Clinic IRB and all patients signed informed consent prior to enrolling in the registry [13]. Patients with stage I-IV disease with serous, endometrioid, clear cell, or mixed histology epithelial carcinomas were included in this analysis. Patients who did not start platinum and taxane based chemotherapy within eight weeks of their primary surgical debulking were excluded.

### Prognostic factors

Demographics and known prognostic markers, as well as epidemiologic exposure variables, were abstracted from the medical record as part of the registry and collected via questionnaire as part of a long-standing case-control study [13]. Variables from previously published prognostic indices that were not part of the standard variable collection for the registry were abstracted as available from the clinical record. Variables included in this analysis were: age at diagnosis, BMI, histology, grade, stage, extent of debulking (no residual disease, ≤1 cm residual, or >1 cm residual tumor), presence of ascites, ASA score, Eastern Oncology Cooperative Group performance status (ECOG PS), surgical complexity score [14], oral contraceptive use, post-menopausal status, number of pregnancies, BRCA1/2 carrier status, family history of ovarian or breast cancer, preoperative albumin, alkaline phosphatase, platelets, hemoglobin, and CA125.

### Statistical methods

The primary outcomes assessed were OS and PFS. OS was defined as time from diagnosis to death due to any cause or last follow-up in patients still alive. PFS was defined as time from diagnosis to the earliest of disease progression, recurrence, re-treatment, death due to any cause, or last follow-up in patients still alive without progression. Prognostic scores from previously published prognostic indices and risk scores were calculated as described in their respective publications. Cause of death was not available in our cohort, thus overall survival was used as a surrogate for disease-specific survival in relevant analyses. Discrete outcome endpoints (e.g. overall survival at 5 years) for nomograms were analyzed using logistic regression. Assessments of previously published models were performed on cases with complete data on predictors; for discrete event endpoints, an event prior to the time-point was considered a failure, patients with sufficient follow-up and without an event at the landmark time point were successes. Patients without sufficient follow-up were considered unevaluable for the endpoint and excluded. Univariate survival assessment of continuous outcomes in the Mayo Clinic cohort was analyzed with Cox proportional hazards regression with Kaplan-Meier curves for graphical examination. Left truncation with the date of registry consent was used in all Cox models to account for any patients enrolling in the registry at the time of recurrence. Prognostic significance was assessed using calibration plots and concordance indices to assess the performance and accuracy of previously published indices when applied to our patient set [15]. Calibration refers to the ability of a model to generate accurate predictions while the c-statistic measures the ability of a model to discriminate levels of risk [16].

## Results

### Patient characteristics

**Figure 1:**
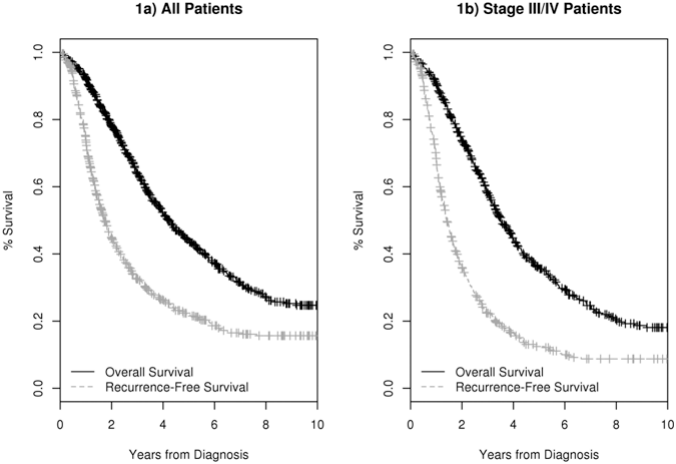
Overall survival and progression-free survival Kaplan Meier curves for Mayo Clinic cohort

A total of 701 patients met inclusion criteria. Patient characteristics are described in Table 1. The median age at diagnosis was 62 years (range 21-87). Approximately half (51%) of patients had no residual disease after primary debulking surgery, with 88% of patients undergoing an optimal debulking procedure, defined as the largest residual tumor nodule measuring less than 1 cm; 12% had greater than 1 centimeter of residual disease [17, 18]. At a median follow-up of 42 months, 384 (55%) patients had died and another 107 (15%) were alive with recurrence. Kaplan Meier estimates for PFS at 12 months (PFS12) and OS at 5 years (OS5) were 73% (95% CI: 70-77) and 45% (95% CI: 41-49) (Figure 1a) respectively, and 68% (95% CI: 64-72) and 36% (95% CI: 32-41) in advanced stage patients (Figure 1b), respectively. 500 patients (71%) had sufficient follow-up to assess 5-year OS in published prognostic models.

### Assessment of previously published models

A summary of the variables included in each model is given in Table 3. Three models for advanced stage disease were assessed. The Chi model was developed to predict overall survival after primary surgery for FIGO stage IIIC EOC and is based on six variables [7]. Data were available to fit the nomogram on 231 (73%) patients in our cohort. The model had an overall c-statistic of 0.695; the calibration curve can be found in figure 3a. The PIEPOC was developed for FIGO stage III-IV EOC [6]. Data were available to fit the model on 357 patients (63%) of which 248 could be evaluated for the 5 year endpoint. A Kaplan- Meier curve showing OS by PIEPOC score (high, medium, low) is shown in figure 3b. The overall c- statistic was 0.574 with very few patients having intermediate (22%) or high risk disease (5%), figure 3c. The Gerestein model was developed to predict 5-year progression-free and overall survival for stage IIB- IV EOC [8]. Data were available to fit the overall survival model on 344 (75%) patients in our cohort. The model had an overall c-statistic of 0.639; the calibration curve can be found in figure 3d.

**Figure 2:**
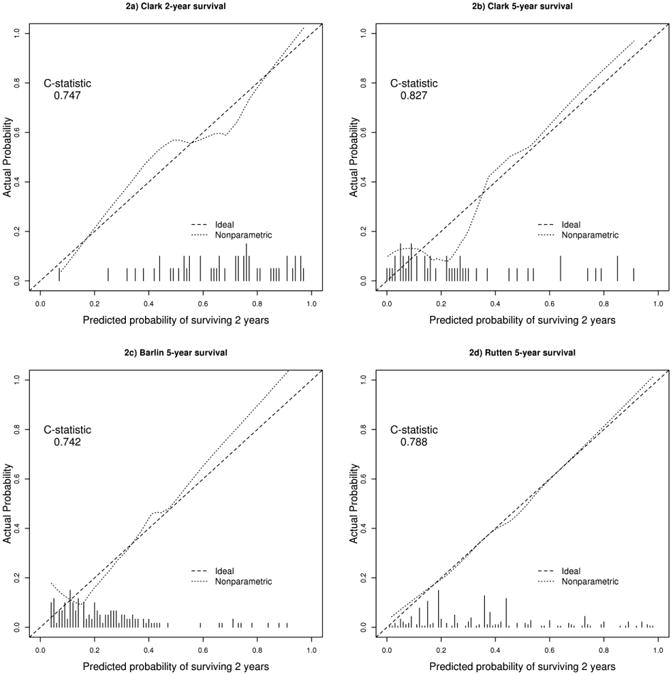
Calibration curves for all-stage models. The x-axis denotes the predicted probability of achieving the endpoint from the model. The y-axis denotes the observed probability of achieving the endpoint in the Mayo dataset. The dotted line is a smoothed fit of the mean model predicted probabilities vs. the mean actual probabilities over a window of predicted probabilities in the Mayo dataset. The dashed identity line denotes optimal calibration. The histogram at the bottom of the plot denotes frequencies of predicted probabilities when the model is fit to the Mayo dataset. a) Clark overall survival at 2 years b) Clark overall survival at 5 years c) Barlin overall survival at 5 years d) Rutten overall survival at 5 years

Three all-stage models were assessed in our cohort of patients. The Clark nomogram was developed to predict overall survival based on 9 variables [4]. The calibration plots for OS at 2 (OS2) and 5 years are shown in figures 2a and 2b. The model c-statistics in our cohort were 0.747 and 0.827 for OS2 and OS5, respectively. Data were only available to fit the model on 49 (10%) patients in our cohort. The Barlin model was developed to predict 5-year disease-specific mortality after primary surgery for epithelial ovarian cancer [5]. Data were available to fit the nomogram on 140 (28%) patients. The model had an overall c-statistic of 0.742; the calibration curve can be found in figure 2c. Lastly, we assessed the validity of the Rutten et al. model in our patient population [10]. For application of the model, family history was used as a surrogate for positive BRCA mutation status in patients with unknown BRCA status. Data were available for 263 (53%) patients. The model had a c-statistic of 0.788; the calibration curve can be found in figure 2d.

### Modeling of outcomes in Mayo Clinic cohort

We also examined predictors of outcome in our cohort. Univariate predictors of inferior overall survival in patients of any stage were age at diagnosis, serous histology, high grade tumor, advanced stage, suboptimal debulking, presence of ascites, higher ASA score, higher ECOG PS, post-menopausal status, increasing number of pregnancies, lower pre-operative albumin, higher pre-operative CA-125, higher pre-operative platelets, and lower pre-operative hemoglobin (all p<0.05, Table 2). Oral contraceptive use was associated with improved overall survival (HR=0.73, p=0.0033). There was no association between BMI and survival (p=0.78). There was a moderate association between self-reported family history of breast or ovarian cancer and improved OS (HR=0.80, p=0.079) and a similar association in BRCA carriers (HR=0.75, p=0.33) although numbers were small (N=130) for BRCA carrier status in this cohort. The most discriminatory univariate predictors of OS were extent of surgical debulking (c-statistic = 0.657, p<0.0001), stage (c-statistic = 0.629, p<0.0001), and presence of ascites (c-statistic = 0.610, p<0.0001).

Similarly, univariate predictors of inferior PFS in patients of any stage were age at diagnosis, serous histology, high grade tumor, advanced stage, suboptimal debulking, presence of ascites, higher ASA score, higher ECOG PS, post-menopausal status, increasing number of pregnancies, lower pre-operative albumin, higher pre-operative CA-125, higher pre-operative platelets, and lower pre-operative hemoglobin (all p<0.05, Table 2). Oral contraceptive use was not significantly associated with improved PFS (HR=0.87, p=0.17). There was no association between BMI and PFS (p=0.27). There was no association between self-reported family history of breast or ovarian cancer (HR=0.99, p=0.93) or BRCA carriers (HR=0.90, p=0.67) and improved PFS. Notably, tumor-related characteristics such as histological subtype, grade, CA-125, and stage were more strongly associated with PFS than OS, while patient- related characteristics such as age and performance status were more strongly associated with OS than with PFS. As with OS, the most discriminatory predictors of PFS were stage (c-statistic = 0.641, p<0.0001), extent of surgical debulking (c-statistic = 0.631, p<0.0001), and presence of ascites (c-statistic = 0.606, p<0.0001).

### Assessment of variables after accounting for stage and debulking

FIGO stage and extent of debulking are well established clinical factors associated with outcome [19-21]. As expected, debulking was included all of the evaluated prognostic models (Table 3) and stage was included in all of the all-stage models. In addition, stage and debulking were the two strongest univariate predictors in our dataset. Therefore, we also assessed the prognostic ability of other variables after accounting for stage and debulking in the outcome models. In all-stage models, the baseline model with stage and debulking was strongly associated with OS (p=3.29×10^−32^, c-statistic = 0.696) and PFS (p= 7.65×10^−42^, c-statistic=0.684). The only variables to add significant prognostic information beyond stage and debulking in both OS and PFS were presence of ascites (OS HR=1.37, 95% CI: 1.06-1.77, p=0.017, model c-statistic=0.715; PFS HR=1.27, 95% CI: 1.01-1.58, p=0.040, model c- statistic=0.704) and ASA score (OS HR=1.47, 95% CI: 1.11-1.95, p=0.0064, model c-statistic=0.712; PFS HR=1.28, 95% CI: 1.01-1.63, p=0.044, model c-statistic=0.700), though ASA score was only available on a subset of patients (n=440) (Supplemental Table 1).

## Discussion

There is considerable interest in developing a prognostic model for ovarian cancer that can be used in the clinic for predicting survival outcomes. Several multivariable models have been put forward in the literature, each with its own set of strengths and weaknesses. In this paper, we used a prospectively ascertained cohort of 701 women with ovarian, fallopian, or peritoneal cancer from our institution as an external validation set for the previously published models. The models assessed showed a wide range of prognostic validity, risk calibration, and missing data when applied to our dataset. We also assessed the prognostic significance of clinical variables in our own population.

**Figure 3:**
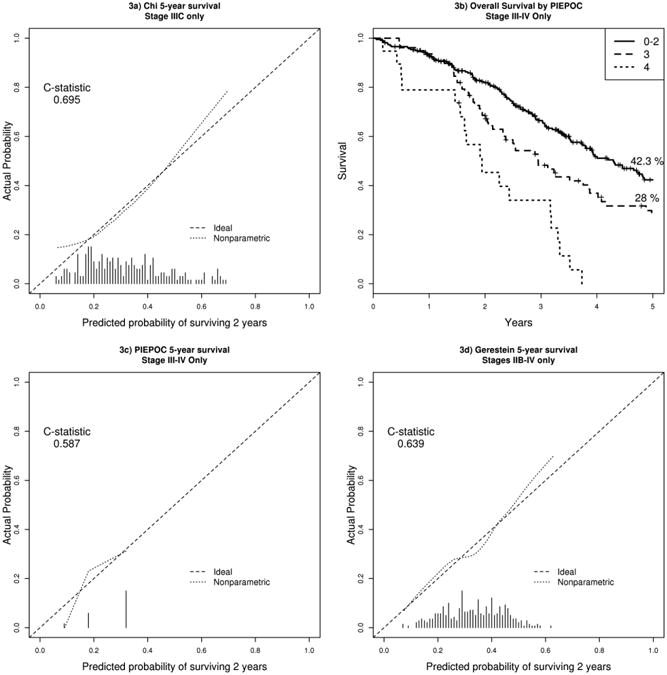
Calibration curves for advanced stage models. The x-axis denotes the predicted probability of achieving the endpoint from the model. The y-axis denotes the observed probability of achieving the endpoint in the Mayo dataset. The dotted line is a smoothed fit of the mean model predicted probabilities vs. the mean actual probabilities over a window of predicted probabilities in the Mayo dataset. The dashed identity line denotes optimal calibration. The histogram at the bottom of the plot denotes frequencies of predicted probabilities when the model is fit to the Mayo dataset. a) Chi overall survival at years 5 b) PIEPOC Kaplan Meier curve for overall survival c) PIEPOC overall survival at years 5 d) Gerestein overall survival at years 5

Given that the majority of women with ovarian cancer are diagnosed at advanced stage, we were particularly interested in models that focused on this group. The model proposed by Chi et. al. is based on patients with stage IIIC disease [7]. The model included only women who underwent a primary cytoreductive surgery, followed by platinum based chemotherapy. This model utilizes six readily available variables (age, grade, histology, platelet count, ascites, and residual disease). We tested this model in 231 women with stage IIIC disease. As seen in figure 3a, our sample set had a good range of predicted probabilities for the model. It did appear to underestimate 5-year OS at the ends of the spectrum (both poor and good prognosis), however, the majority of our cohort fell in the middle range where calibration was best. Overall, the model had a c-statistic of 0.695 with good calibration across the range of estimates.

In 2007, Teramukai et. al. published the PIEPOC prognostic index. This model uses four prognostic factors (age, performance status (PS), histology, and residual disease) to classify patients into three risk groups derived from regression analysis [6]. The data required for this model was available in 248 women with stage III/IV disease in our sample set. The three risk groups do stratify for outcome, as seen in the original cohort, however there were very few patients that fell in the intermediate (22%) and high (5%) risk disease groups. It is important to note, however, the difference in OS5 between the PIEPOC cohort (61%, 40% and 16% from low to high risk res) and our cohort (42.3%, 28%, and 0%), indicating a lack of calibration in our cohort. The majority of our sample set was in the low risk group, primarily due to the high rate of tumor debulking to less than 1 cm, and therefore we are only able to make limited conclusions regarding the intermediate and high risk groups. This model has also been evaluated using data from the Cancer Research UK Edinburgh Ovarian Cancer Database (n=894) [12]. As in our dataset, the OS5 in that dataset was significantly lower than in the Japanese cohort [12]. This suggests that the utility of this model in predicting OS may be less in a Caucasian population.

Lastly for advanced stage models, we applied the Gerestein model to our dataset. This model predicts 5- year PFS and OS in women who had completed a primary cytoreductive surgery followed by platinum- taxane based chemotherapy for stage IIB- IV ovarian cancer [8]. This model takes into account only 1-2 additional clinical factors besides residual tumor burden (platelet count for PFS5 and platelet count along with hemoglobin for OS5). These data were available for 75% of our stage IIB-IV patients. As seen in figure 3d, this model had good calibration along the entire range of estimates with an overall statistic of 0.639. Because of the small number of accessible variables required for this model, the majority of our stage IIB-IV patients were included in this validation set. This suggests ease of clinical use, since the factors included in the model are readily available. The combination of the good calibration and c- statistic also suggest that this model may be of clinical use.

Recently, van de Laar and colleagues used a multi-institutional database in the Netherlands to identify a group of 542 women with advanced stage epithelial ovarian cancer to use as an external validation set for the Chi, Gerestein and PIEPOC models [9]. All three models showed general applicability and reasonable to moderately good c-indices. Compared to FIGO staging alone, all showed better accuracy. As noted with our external validation set, due to the number of variables in the models, the actual number of samples in the validation cohort significantly drops, most notably when using models with numerous or less commonly assessed variables. Our study here also confirms the conclusion that the three models showed general applicability and a reasonably well predictive performance. In the current study, our data also extends to early stage disease.

For models that included women with early stage disease, we evaluated the Clark, Barlin and Rutten models [4, 5, 10]. The Clark model did have excellent concordance in our cohort (c-statistics of 0.747 and 0.827 for OS2 and OS5 respectively), however this model may be limited clinically by the variables required for its use. In our large sample set, only 49 of 500 (10%) women had all 9 variables collected and 5 years of follow-up. In our cohort, alkaline phosphatase was not routinely collected perioperatively and was the primary limiting factor. Overall, the small sample size available limits our ability to truly assess this model using our Mayo patient cohort. Another limiting factor in the Clark model is the definition of residual disease used in this model. In this model, residual disease is classified as < 2cm, 2-5 cm, or >5 cm which is not consistent with the currently clinically accepted classification: no macroscopic disease, < 1cm, or > 1 cm residual disease categories [18, 22, 23]. Also, approximately 10% of the chemotherapy regimens given in patients used to design the Clark model were not platinum based regimens. These factors may play a role in the moderate to poor calibration (figure 2a-b) and limit its clinical use.

The Barlin model is a user-friendly seven-variable nomogram developed to predict 5-year disease- specific mortality for all stages of epithelial ovarian cancer [5]. Analysis using our sample set did result in a strong c-statistic (0.742); however the calibration was weak between the 5-year survival probabilities of 0-40%, which is where the majority of our patients fell. The majority of the patients used to develop the model had stage IIIC disease, and therefore it appears that overall, our “poor prognosis” patients (advanced stage disease) out-performed the predicted survival from the Barlin model, i.e. their actual risk was lower than predicted risk. In the early stage/good prognosis patients, our sample size is very small and therefore it is very difficult to make any definitive conclusions.

A more recent all stage prognostic model has been proposed by Rutten et. al. [10]. Unlike the other models assessed, their cohort of women includes those who have undergone an interval debulking surgery (52% of their cohort). It should be noted that our cohort includes only women who had primary debulking. Despite this difference in the patient population, the calibration using this model was the strongest of all models tested. Ascites did not appear as a prognostic variable in their model, however, it should be noted that only 18% of their patients had ascites whereas in our cohort 57% had ascites. The definition for ascites used in the Rutten model was fluid greater than 500 mL; in our data ascites was only defined in the medical record as present or absent. This may account for the difference in the strength of ascites as a prognostic variable.

In addition to validation of published models, we also examined predictors of outcome in the Mayo cohort. On univariate analysis, numerous variables impacted OS and PFS (table 1). Many of these variables were included in some of the aforementioned models. Interestingly, use of oral contraceptives, known to reduce the incidence of ovarian cancer [24], was linked to an improved OS following a diagnosis of ovarian cancer. Also of note, in our data set there was no association between BMI and OS.

It is well established that FIGO stage and extent of initial cytoreductive surgery are key prognostic variables. We confirmed this in our cohort and sought to evaluate the impact of other variables after taking stage and debulking into account. Only presence of ascites and ASA score provided additional prognostic information beyond stage and debulking, though with caveats that ascites was of moderate significance (p=0.017 for OS and p=0.040 for PFS) and ASA score was unavailable in 37% of patients. Thus we did not propose a new clinical model from our dataset. The lack of strong predictors beyond stage and debulking may explain the wide range of variables in the published models we assessed. This also highlights the need for new prognostic biomarkers in this cancer from other sources, such as molecular profiling. Finally, inclusion of stage and debulking is recommended (and sufficient at this time) when adjusting for clinical prognostic factors in the evaluation of new outcome models and other time to event models in ovarian cancer.

Strengths of our study include the use of a large, prospectively ascertained cohort of women with ovarian, tubal and peritoneal cancer, who were treated in a standardized fashion and followed long- term for recurrence and survival. To our knowledge, this study is the first to attempt to validate the major prognostic models for both advanced and all-stage disease. Our ability to assess two of the models, namely the Clark and Barlin models, was limited because we do not routinely collect alkaline phosphatase and albumin unless clinically indicated, and so had incomplete data on which to run the models. To be considered useful, a prognostic model must not only show external validation, but also clinical usefulness and effectiveness. Although no impact studies have been done using these models, accessibility of some of the variables and cut-offs likely restrict their clinical utility.

In summary, models for ovarian cancer in the published literature generally had adequate replication in external data, in our study as well as prior validation studies in the advanced stage models [9, 12], though application of the models may be limited due to missing or unavailable data. The recently published Rutten model appears to have the best clinical utility in our center's data, but clinicians and patients should utilize multiple models for which they have the available data and compare results for a range of estimates. Analysis of prognostic factors in our dataset again reinforces the fact that the most important prognostic features in EOC are stage and debulking status. The addition of other variables such as ascites may provide additional prognostic information, but the impact of additional variables beyond stage and debulking have diminishing returns in prognostic prediction.

## Supplementary Material

supplement Supplemental Table 1. Summary of single variable prognostic models (overall survival and progression- free survival) after adjusting for stage and debulking, all stages

## Figures and Tables

**Table 1:** Patient characteristics of Mayo Cohort

Variable		N	%

Histology	Serous	547	78.0%
	Endometrioid	79	11.3%
	Clear Cell	37	5.3%
	Mixed/other	38	5.4%

Grade	Low (1-2)	82	12.2%
	High (3-4)	589	87.8%

Stage	I (substage unknown)	1	0.1%
	IA	20	2.9%
	IB	3	0.4%
	IC	57	8.2%
	II (substage unknown)	3	0.4%
	IIA	3	0.4%
	IIB	6	0.9%
	IIC	37	5.3%
	III (substage unknown)	11	1.6%
	IIIA	5	0.7%
	IIIB	21	3.0%
	IIIC	418	60.3%
	IV (substage unknown)	106	15.3%
	IVC	2	0.3%

Extent of Disease after Primary Debulking Surgery	No residual disease	342	50.9%
	0-1 cm	250	37.2%
	>1 cm	80	11.9%

Ascites	Absent	267	43.3%
	Present	350	56.7%

ASA Score	1-2	272	61.8%
	3-4	168	38.2%

ECOG PS	0	334	75.1%
	1	90	20.2%
	2-4	21	4.7%
Surgical Complexity Score	Low (0-3)	59	13.2%
	Intermediate (4-7)	251	56.3%
	High (8+)	136	30.5%

Oral contraceptive Use	No	247	38.5%
	Yes	395	61.5%
Post Menopausal	No	139	22.0%
	Yes	493	78.0%

Number of pregnancies	0	86	13.4%
	1	45	7.0%
	2	162	25.2%
	3+	349	54.4%
BRCA 1/2 Carrier	No	94	72.3%
	Yes	36	27.7%

Family History of Breast and/or Ovarian Cancer	No	499	76.7%
	Yes	152	23.4%

BMI	Normal/Underweight (<25)	250	39.5%
	Overweight (25.0-29.9)	200	31.6%
	Obese (30+)	183	28.9%

**Continuous Variables**		**N**	**Median (range)**

Albumin, g/dL		327	4.0 (2.1-5.0)

Age, years		701	62 (21-87)

CA125 pre-surgery		532	493 (2-83,399)

BMI, kg/m2		633	27 (16-51)

Alkaline phosphatase, U/L		164	91.5 (26-615)

Platelets, ×10^9^/L		505	338 (59-1,096)

Hemoglobin, mmol/L		505	7.9 (4.8-10.1)

**Table 2:** Summary of unadjusted prognostic variables (overall survival and progression-free survival), all stages

		N	OS HR	95% CI	p-value	c-index	PFS HR	95% CI	p-value	c-index
Morphology	Serous	54	ref			0.551	Ref			0.568
	Endometrioid	79	0.33	0.21-	<0.00		0.21	0.14	<0.00	
	Clear Cell	37	0.56	0.31 -	0.048		0.49	0.-29	0.0079	
	Mixed/other	38	0.61	0.37-	0.053		0.54	0.-35	0.0078	
Grade	Low	82	ref			0.540	Ref			0.550
	High	58	2.24	1.50-	<0.00		3.05	2.10	<0.00	
Stage	I	81	ref			0.629	Ref			0.641
	II	49	2.54	1.05-	0.039		2.49	1.19	0.016	
	III	45	7.95	3.93-	<0.00		8.96	5.-03	<0.00	
	IV	10	12.78	6.17-	<0.000		14.24	7.79	<0.000	
Debulking	No residual disease	34	ref			0.657	Ref			0.631
	0-1 cm	25	2.81	2.23-	<0.00		2.86	2.33	<0.00	
	>1 cm	80	3.48	2.57-	<0.000		2.83	2.12-	<0.000	
Ascites	Absent	26	ref			0.610	Ref			0.606
	Present	35	2.36	1.86-	<0.00		2.28	1.86	<0.00	
ASA Score	1-2	27	ref			0.591	Ref			0.574
	3-4	16	1.98	1.52-	<0.00		1.78	1.41	<0.00	
ECOG PS	0	33	ref			0.559	Ref			0.531
	1	90	1.35	0.97-	0.079		1.17	0.88	0.28	
	2-4	21	3.26	2.04-	<0.00		1.96	1.25	0.0033	
Surgical Complexity	Low (0-3)	59	ref			0.558	Ref			0.571
	Intermediate (4-7)	25	0.58	0.40-	0.0057		0.75	0.53	0.12	
	High (8+)	13	0.98	0.65-	0.91		1.37	0.-95	0.092	
OC Use	No	24	ref			0.542	Ref			0.514
	Yes	39	0.73	0.59-	0.0033		0.87	0.72	0.17	
Post Menopausal	No	13	ref			0.538	Ref			0.528
	Yes	49	1.56	1.18-	0.0019		1.51	1.17	0.0015	
Number of	0	86	ref			0.538	Ref			0.539
	1	45	1.24	0.73-	0.42		1.15	0.72	0.56	
	2	16	1.76	1.20-	0.0036		1.64	1.17	0.0046	
	3+	34	1.54	1.08-	0.017		1.50	1.10	0.011	
BRCA 1/2 Carrier	No	94	ref			0.536	Ref			0.513
	Yes	36	0.75	0.41-	0.33		0.90	0.57	0.67	
Family History	No	49	ref			0.519	Ref			0.503
	Yes	15	0.80	0.62-	0.079		0.99	0.79	0.93	
BMI	Normal/Underweight	25	ref			0.515	Ref			0.519
	Overweight (25.0-	20	1.04	0.80-	0.79		1.17	0.93	0.18	
	Obese (30+)	18	1.01	0.78-	0.95		1.18	0.94	0.16	
Albumin, log2		32	0.40	0.22-	0.0029	0.596	0.26	0.14	<0.00	0.592
Age, per 10 years		70	1.26	1.15-	<0.00	0.573	1.21	1.12	<0.00	0.553
CA125 pre-surgery,		53	1.08	1.03-	0.0008	0.559	1.11	1.06	<0.00	0.577
BMI		63	1.00	0.98-	0.78	0.499	1.01	0.99	0.27	0.514
Alk phosphate,		16	1.33	0.93-	0.11	0.574	1.14	0.83	0.42	0.538
Platelets, per 100		50	1.16	1.08-	<0.00	0.576	1.13	1.05	0.0005	0.572
Hemoglobin		50	0.84	0.73-	0.012	0.552	0.82	0.73	0.0008	0.554

**Table 3:** Comparison of variables in published models and nomograms

	Model					
Prognostic factor	Clark (All stages) N=49	Barlin (All stages) N=140	Rutten (all stage) N=263	Chi (Stage IIIC) N=231	PIEPOC (Stage III-IV) N=357	Gerestein (Stage IIB-IV) N=344
FIGO stage	In final model	In final model	In final model	Not applicable	Assessed, not in final model	Assessed, not in final model
Residual disease (debulking)	In final model	In final model	In final model	In final model	In final model	In final model
Grade	In final model	Assessed, not in final model	Not assessed	In final model	Assessed, not in final model	Not assessed
Histology	In final model	In final model	In final model	In final model	In final model	Assessed, not in final model
Ascites	In final model	Assessed, not in final model	In final model	In final model	Not assessed	Assessed, not in final model
Age	In final model	In final model	In final model	In final model	In final model	Assessed, not in final model
Performance status	In final model	Assessed, not in final model	In final model	Not assessed	In final model	Assessed, not in final model
Alkaline phosphatase	In final model	Assessed, not in final model	Not assessed	Not assessed	Not assessed	Not assessed
Albumin	In final model	In final model	Assessed, not in final model	Not assessed	Not assessed	Assessed, not in final model
Family history suggestive of HBOC	Not assessed	In final model	Not assessed	Not assessed	Not assessed	Not assessed
ASA status	Not assessed	In final model	Assessed, not in final model	Not assessed	Not assessed	Not assessed
Platelet count	Not assessed	Assessed, not in final model	Not assessed	In final model	Not assessed	In final model
Hemoglobin	Not assessed	Assessed, not in final model	Not assessed	Not assessed	Not assessed	For OS5 only
Primary or interval debulking	Not assessed	Not assessed	In final model	Not assessed	Not assessed	Not assessed
BRCA status	Not assessed	Not assessed	In final model	Not assessed	Not assessed	Not assessed

In final model = Variable was included in original authors' final model

Assessed, not in final model = Variable was assessed in original authors' analysis but was not included in the final model

Not assessed = Variable was not assessed in original authors' analysis.

For OS5 only = Included in the final model for 5 year survival only.

Not applicable = Variable does not apply to patients in the analysis

## Highlights

- Previously published ovarian cancer prognostic markers show a range of external validity and calibration
- First external validation of the recently published Rutten model
- Stage and extent of initial debulking remain the overriding variables in predicting outcome in ovarian cancer
