# Supplementary material for: Assessment of published models and prognostic variables in epithelial ovarian cancer at Mayo Clinic
Source: Gynecol Oncol. Author manuscript; Available in PMC 2016 Apr 1. (PMC4380608; doi:10.1016/j.ygyno.2015.01.539)
Supplement: supplement — al Table 1. Summary of single variable prognostic models (overall survival and progression- free survival) after adjusting for stage and debulking, all stages [file NIHMS662821-supplement.pdf]

Supplemental Table 1

Multivariable model of Stage and Debulking									
		**OS HR**	**95% CI**	**p-value**	**c-index**	**PFS HR**	**95% CI**	**p-value**	**c-index**
Stage	I	ref			0.696	Ref			0.684
	II	2.23	0.92-5.38	0.075		2.06	0.97-4.35	0.060	
	III	4.78	2.32-9.84	<0.0001		5.95	3.29-10.74	<0.0001	
	IV	7.54	3.55-15.98	<0.0001		9.32	5.01-17.33	<0.0001	
Debulking	No residual disease	ref				Ref			
	0-1 cm	2.00	1.57-2.55	<0.0001		1.89	1.53-2.33	<0.0001	
	>1 cm	2.54	1.86-3.48	<0.0001		1.86	1.39-2.50	<0.0001	
**Prognostic Performance of Variables After Adjustment for Stage and Debulking**									
		**OS HR**	**95% CI**	**p-value**	**Model c-index**	**PFS HR**	**95% CI**	**p-value**	**Model c-index**
Morphology	Serous	ref			0.700	Ref			0.686
	Endometrioid	0.79	0.50-1.26	0.32		0.57	0.35-0.90	0.016	
	Clear Cell	2.13	1.15-3.94	0.017		2.19	1.23-3.89	0.0079	
	Mixed / other	0.79	0.47-1.32	0.37		0.87	0.55-1.37	0.56	
Grade	Low	ref			0.700	ref			0.689
	High	1.30	0.86-1.96	0.22		1.66	1.13-2.44	0.010	
Ascites	Absent	ref			0.715	ref			0.704
	Present	1.37	1.06-1.77	0.017		1.27	1.01-1.58	0.040	
ASA Score	1-2	ref			0.712	ref			0.700
	3-4	1.47	1.11-1.95	0.0064		1.28	1.01-1.63	0.044	
ECOG PS	0	ref			0.715	Ref			0.698
	1	1.29	0.92-1.80	0.14		1.10	0.82-1.47	0.52	
	2-4	2.83	1.75-4.57	<0.001		1.56	0.98-2.47	0.059	
Surgical Complexity Score	Low (0-3)	ref			0.700	Ref			0.693
	Intermediate (4-7)	0.76	0.51-1.13	0.18		0.94	0.65-1.37	0.76	
	High (8+)	0.83	0.54-1.28	0.39		1.06	0.72-1.57	0.77	
OC Use	No	ref			0.703	Ref			0.692
	Yes	0.81	0.65-1.01	0.061		0.95	0.78-1.16	0.63	
Post Menopausal	No	ref			0.699	Ref			0.692
	Yes	1.14	0.85-1.53	0.38		1.10	0.84-1.43	0.48	
Number of pregnancies	0	ref			0.703	Ref			0.702
	1	1.15	0.67-1.97	0.61		1.02	0.64-1.64	0.93	
	2	1.60	1.08-2.37	0.020		1.28	0.90-1.81	0.17	
	3+	1.28	0.89-1.84	0.18		1.11	0.81-1.52	0.53	
BRCA 1/2 Carrier	No	ref			0.738	Ref			0.703
	Yes	0.72	0.40-1.30	0.28		0.76	0.47-1.22	0.24	
Family History	No	ref			0.699	Ref			0.693
	Yes	0.77	0.59-0.99	0.045		0.88	0.70-1.11	0.29	
BMI	Normal/Underweight (<25)	ref			0.699	Ref			0.700
	Overweight (25.0-29.9)	0.97	0.74-1.27	0.84		1.22	0.96-1.56	0.10	
	Obese (30+)	0.92	0.70-1.19	0.52		1.12	0.89-1.42	0.34	
Albumin, log2		0.75	0.38-1.50	0.42	0.710	0.43	0.23-0.81	0.0093	0.704
Age, per 10 years		1.16	1.05-1.28	0.0036	0.705	1.09	1.00-1.19	0.055	0.686
CA125 pre-surgery, log2		0.96	0.91-1.01	0.14	0.703	0.99	0.95-1.05	0.84	0.691
BMI		0.99	0.97-1.01	0.15	0.703	1.00	0.98-1.01	0.90	0.693
Alk phosphate, natural log		1.07	0.72-1.57	0.75	0.697	0.78	0.55-1.12	0.19	0.679
Platelets, per 100 count		1.05	0.96-1.14	0.30	0.706	0.99	0.92-1.07	0.86	0.694
Hemoglobin		0.96	0.83-1.10	0.53	0.707	0.92	0.81-1.05	0.22	0.706
